# A year in pharmacology: new drugs approved by the US Food and Drug Administration in 2022

**DOI:** 10.1007/s00210-023-02465-x

**Published:** 2023-03-23

**Authors:** Gizem Kayki-Mutlu, Zinnet Sevval Aksoyalp, Leszek Wojnowski, Martin C. Michel

**Affiliations:** 1 Department of Pharmacology, Faculty of Pharmacy, Ankara University, Ankara, Turkey; 2 Department of Pharmacology, Faculty of Pharmacy, Izmir Katip Celebi University, Izmir, Turkey; 3 Department of Pharmacology, University Medical Center, Universitätsmedizin Mainz, Johannes Gutenberg University, Langenbeckstr. 1, 55118 Mainz, Germany

**Keywords:** FDA, New drugs, First-in-class, Next-in-class

## Abstract

While new drug approvals by the U.S. Food and Drug Administration (FDA) had remained stable or even increased in the first 2 years of the COVID-19 pandemic, the 37 newly approved drugs in 2022 are considerably less than the 53 and 50 new drugs approved in 2020 and 2021, respectively, and less than the rolling 10-year average of 43. As in previous years of this annual review, we assign these new drugs to one of three levels of innovation: first drug against a condition (“first-in-indication”), first drug using a novel molecular mechanism (“first-in-class”), and “next-in-class,” i.e., a drug using an already exploited molecular mechanism. We identify two “first-in-indication” (ganaxolon and teplizumab), 20 (54%) “first-in-class,” and 17 (46%) “next-in-class” drugs. By treatment area, rare diseases and cancer drugs were once again the most prevalent (partly overlapping) therapeutic areas. Other continuing trends were the use of accelerated regulatory approval pathways and the reliance on biopharmaceuticals (biologics).

## Introduction

The U.S. Food and Drug Administration (FDA) has approved 37 new molecular entities in 2022 (U. S. Food and Drug Administration 2023). This is less than the 53 and 50 new drug approvals in 2020 and 2021, respectively (Kayki-Mutlu and Michel 2021; Kayki-Mutlu et al. 2022) and less than the rolling 10-year average of 43 approvals per year. In continuation of our similar analyses for 2020 and 2021, we here review the degree of pharmacological innovation in the newly approved compounds in 2022. Discussing the specific advantages and disadvantages of individual compounds against their competitors (best-in-class, best-in-indication) is beyond the scope of this article and should be left to experts in a therapeutic area. Similarly, drug pricing, particularly in oncology, and how it relates to the clinical benefit/risk profile will not be discussed, due to the complexity of the issue and the requirement for specific expertise in a therapeutic area; this a task typically reserved for Health Technology Assessment bodies in various countries, such as the National Institute for Health and Care Excellence in the UK. Based on these data, we discuss emerging trends in drug approvals.

## Methods

Our approach is identical to that used in previous annual updates of newly approved drugs (Kayki-Mutlu and Michel 2021; Kayki-Mutlu et al. 2022): our analyses are based on the list of new molecular entities approved by the FDA in 2022 as communicated by the agency (U. S. Food and Drug Administration 2023). We did not include vaccines, generics, or generic versions of biopharmaceuticals (“biosimilars”), or already approved drugs that received marketing authorization for one or more additional indications and/or in a novel formulation; newly approved drug combinations were only considered if at least one of the combination partners is a novel chemical or biopharmaceutical entity (mostly therapeutic antibodies). Of note, other regulatory agencies may have approved the same compounds earlier than the FDA, may do so at later points in time, may choose not to approve some of these compounds, or may choose to approve compounds not approved by the FDA. Such differences may at least partly reflect that originator companies may not have filed for approval in all jurisdictions, at least not at the same time. Furthermore, the time from filing to approval may have been longer or shorter with the FDA compared to other regulatory agencies. Our focus on FDA approvals does not imply any opinion on the scientific quality of approvals by the FDA as compared to the regulatory authorities in other jurisdictions but rather uses the FDA as a point of reference, due to its status as one of the most influential drug regulatory authorities. All indications refer to adults unless specifically noted otherwise.

## Oncology

Melanoma is a common skin cancer with increasing incidence and high mortality (Siegel et al. 2022). A combination of nivolumab with relatlimab-rmbw has been approved for unresectable or metastatic melanoma in adult and pediatric patients. Resulting from a priority review, nivolumab/relatlimab constitutes a first-in-class dual immunotherapy. The two human immunoglobulin G4 (IgG4) monoclonal antibodies in this combination are immune checkpoint inhibitors. Nivolumab, approved in 2014, targets the programmed cell death-1 (PD-1) receptor on T-cells and blocks the binding of PD ligands to this receptor (Paik 2022b). Hence, nivolumab decreases the inhibition of immune response and stimulates the T-cell proliferation and cytokine synthesis (Paik 2022b). Relatlimab is a monoclonal antibody against lymphocyte-activation gene 3 (LAG-3) receptor in immune cells (Tawbi et al. 2022). LAG-3 has a role in blocking T-cell proliferation and stimulating T-cell exhaustion. Relatlimab blocks the binding of LAG-3 ligands (e.g., MHC II), reduces LAG-3 pathway-mediated immune response inhibition, and restores the effector function of exhausted T-cells (Paik 2022b). Blocking PD-1 and LAG-3 with nivolumab and relatlimab synergistically enhances T-cell activation and antitumor immunity. Nivolumab/relatlimab has a good tolerability profile (Tawbi et al. 2022). The most frequent adverse events are fatigue, rash, pruritus, arthralgia, hypothyroidism, vitiligo, and diarrhea (Paik 2022b). Serious adverse reactions are adrenal insufficiency, pneumonia, acute myocardial infarction, myocarditis, and pneumonitis (Paik 2022b).

Uveal melanoma is a rare and specific form of melanoma that has a dismal prognosis (Martinez-Perez et al. 2021). Glycoprotein 100 (gp100) is strongly expressed in melanoma cells (Wagner et al. 1997), which makes it a new target for the treatment of uveal melanoma. Tebentafusp-tebn (formerly IMCgp100) is a first-in-class, bispecific fusion protein which has two components (Martinez-Perez et al. 2021). A T-cell receptor targets the gp100 protein (Olivier and Prasad 2022). An anti-CD3 effector is its other component and triggers the anti-tumoral T-cell response (Olivier and Prasad 2022). Tebentafusp belongs to T-cell receptor (TCR)-based therapies (Dhillon 2022b). Tebentafusp has been approved for HLA-A*02:01-positive patients with unresectable or metastatic uveal melanoma and granted a breakthrough therapy designation following a priority review (Nathan et al. 2021). The most frequent adverse events observed with tebentafusp are rash, fever, tremor, low blood pressure, itching, and erythema (Dhillon 2022b).

The expression of prostate-specific membrane antigen (PSMA) is elevated in prostate cancer cells, and its form circulating in blood is exploited for diagnostic purposes including monitoring of patients (Jeong and Kwak 2021). Lutetium (^177^Lu) vipivotide tetraxetan is a targeted radiopharmaceutical. Vipivotide tetraxetan binds to PSMA, and beta emission from ^177^Lu causes the DNA damage and death of cancer cells (Keam 2022a). ^177^Lu vipivotide tetraxetan has been approved for PSMA-positive, metastatic, and castration-resistant prostate cancer in patients previously treated with androgen receptor inhibitors and taxane drugs (Aschenbrenner 2022). ^177^Lu vipivotide tetraxetan was granted a breakthrough therapy status based on a priority review. The most frequent adverse events are gastrointestinal complaints, xerostomia, and anemia. Clinically significant adverse effects are severe myelosuppression and renal toxicity (Keam 2022a).

Cholangiocarcinoma has poor prognosis and scarce treatment options (King and Javle 2021). Futibatinib has been approved for unresectable, locally advanced, or metastatic intrahepatic cholangiocarcinoma harboring fibroblast growth factor receptor (FGFR)-2 gene fusions or other rearrangements in patients previously treated with non-FGFR therapies (Goyal et al. 2023). Futibatinib was granted a priority review, accelerated approval, and breakthrough designation. Futibatinib is a small-molecule, potent, selective, irreversible, and third-generation inhibitor of the protein kinases FGFR1-4 (King and Javle 2021; Syed 2022). Futibatinib reduces the proliferation and increases death in tumor cells via blocking the FGFR signaling pathways. The most common adverse events of futibatinib were xerostomia, dryness of the skin and eyes, alopecia, loss of appetite, urinary tract infection, nail toxicity, tiredness, stomatitis, pain, palmar-plantar erythrodysesthesia syndrome, and oral and gastrointestinal complaints (Syed 2022).

Tremelimumab-actl is a monoclonal antibody against the cytotoxic T-lymphocyte-associated antigen 4 (CTLA-4) and acts as an immune checkpoint inhibitor (Comin-Anduix et al. 2016). Tremelimumab prevents the binding of the antigen-presenting cell ligands to CTLA4. In consequence, antigen-presenting cell ligands bind to CD28, another T-cell receptor, and the resultant effect is the T-cell activation (Maker et al. 2005). Tremelimumab in combination with durvalumab (anti-PD-L1 antibody) has been approved for the treatment of unresectable hepatocellular carcinoma. The most common adverse evets are gastrointestinal complaints, rash, fatigue, and myalgia, according to the tremelimumab prescribing information.

Teclistamab-cqyv is the first T-cell-redirecting bispecific IgG4 antibody against both CD3 and B-cell maturation antigen (BCMA) (Moreau et al. 2022). Teclistamab has been approved for relapsed or refractory multiple myeloma in patients who have used more than four previous treatment lines (Kang 2022b). It received a breakthrough designation following a priority review and an accelerated approval. Teclistamab is activated in T-cells and leads to the death of BCMA-expressing myeloma cells (Moreau et al. 2022). The most common adverse events observed with teclistamab are cytokine release syndrome and infection (Moreau et al. 2022).

Epithelial ovarian cancer has a high mortality and is characterized by increased folate receptor alpha expression (O'Shannessy et al. 2013; Marchetti et al. 2014). Mirvetuximab soravtansine-gynx is a first-in-class antibody–drug conjugate approved for folate receptor alpha-positive, platinum-resistant epithelial ovarian cancer in patients who have used one to three systemic treatment options before. It was granted accelerated approval following a priority review. Mirvetuximab soravtansine consists of a humanized folate receptor alpha-binding monoclonal antibody, cleavable disulfide linker, and the potent microtubule inhibitor DM4 (Ab et al. 2015). The antibody–drug conjugate binds the folate receptor alpha and then enters the cells. The blood-stable disulfide linker is cleaved (Moore et al. 2018), and the released DM4 binds to tubulin, which inhibits cell proliferation (Oroudjev et al. 2010). Mirvetuximab soravtansine has a boxed warning about ocular toxicity. The most frequent adverse events are vision impairment, fatigue, nausea, keratopathy, diarrhea, and dry eye (Heo 2023).

The KRAS G12C mutation is common in non-small cell lung cancer (NSCLC) (Reita et al. 2022). Adagrasib is a small molecule, potent, highly selective, and irreversible inhibitor of KRASG12C (Brazel et al. 2022). Adagrasib leads to a KRASG12C inactive state and inhibits tumor cell growth and viability via inhibition of signaling pathways downstream of KRAS (Brazel et al. 2022). Adagrasib is the second approved KRAS inhibitor for NSCLC after sotorasib, which was approved in 2021 (Kayki-Mutlu et al. 2022); adagrasib was found to be superior to sotorasib in the objective response rate (Tian and Yang 2022). Adagrasib has been approved for KRASG12C-mutated, locally advanced, or metastatic NSCLC in patients previously treated with platinum-based chemotherapy and anti–programmed death 1 or programmed death ligand 1 therapy. Adagrasib was granted an accelerated approval and a breakthrough therapy status. The most common adverse events with adagrasib are diarrhea, nausea, fatigue, and vomiting (Janne et al. 2022).

Mosunetuzumab-axgb is a first-in-class, bispecific antibody which bridges cytotoxic T-cells with malignant B-cells to eliminate the latter (Budde et al. 2022; Kang 2022a). One of the two antibody arms binds specifically to the B-lymphocyte antigen CD20 on the surface of malignant B-cells. Via the other antibody arm, cytotoxic T-cells are bound by specific binding to the CD3 antigen. Mosunetuzumab has been approved for relapsed or refractory follicular non-Hodgkin lymphoma in patients previously treated with two or more other systemic therapies. Mosunetuzumab was granted breakthrough and orphan drug designations based on a priority review and accelerated approval (Table 1). Mosunetuzumab has a boxed warning due to the cytokine release syndrome risk; the most frequent adverse events are hypophosphatemia, neutropenia, high fever, and headache (Kang 2022a).

**Table 1:** 2022 FDA orphan drug approvals. The percentage is that of orphan drugs within all drugs approved by the FDA in 2022 taken as 100%. https://www.accessdata.fda.gov/scripts/opdlisting/oopd/listResult.cfm

Orphan drug (54%)	Approved indication
Adagrasib	KRAS G12C-positive non-small cell lung cancer
Anacaulase-bcdb	Debridement of acute, deep dermal burns in hospitalized patients
Futibatinib	Cholangiocarcinoma
Ganaxolone	Cyclin-dependent kinase-like 5 (CDKL5) gene-related early-onset infantile epileptic encephalopathy
Mavacamten	Symptomatic hypertrophic cardiomyopathy
Mirvetuximab soravtansine-gynx	Ovarian cancer
Mitapivat	Pyruvate kinase deficiency
Mosunetuzumab-axgb	Follicular lymphoma
Nivolumab and relatlimab-rmbw	Stage IIb to IV melanoma
Olipudase alfa-rpcp	Acid sphingomyelinase deficiency (Niemann-Pick disease)
Olutasidenib	Acute myeloid leukemia
Pacritinib	Myelofibrosis
Sodium phenylbutyrate and tauroursodeoxycholic acid dihydrate	Amyotrophic lateral sclerosis (ALS); orphan status only for Wolfram syndrome
Spesolimab-sbzo	Generalized pustular psoriasis
Sutimlimab-jome	Autoimmune hemolytic anemia
Tebentafusp-tebn	Uveal melanoma
Teclistamab-cqyv	Multiple myeloma
Terlipressin	Hepatorenal syndrome
Tremelimumab-actl	Hepatocellular carcinoma
Vutrisiran	Transthyretin-mediated amyloidosis (ATTR amyloidosis)

Myelofibrosis is a rare myeloproliferative neoplasm; myelofibrosis patients have an augmented activation of Janus kinase signaling, which leads to severe thrombocytopenia with a poor prognosis (Patel and Odenike 2020; Mascarenhas 2022). Pacritinib is a small-molecule, highly selective inhibitor of Janus kinase-2/interleukin-1 receptor-associated kinase 1 (Singer et al. 2016) that decreased the splenomegaly and improved symptoms in myelofibrosis patients (Mascarenhas 2022). Pacritinib has been approved for intermediate or high-risk myelofibrosis with a thrombocyte level < 50 × 10^9^/L. Pacritinib was granted an accelerated approval following a priority review. Adverse events associated with pacritinib such as anemia, diarrhea, nausea, and edema are controllable (Lamb 2022b).

Acute myeloid leukemia (AML) is a hematologic malignancy and the most common leukemia among adults. Olutasidenib is a potent and selective inhibitor of mutated isocitrate dehydrogenase 1 (IDH1). The incidence of IDH mutations is nearly 20% in AML patients (Issa and DiNardo 2021). Olutasidenib has been approved for relapsed or refractory acute myeloid leukemia with mutated IDH1. Treatment-eligible patients are determined by an FDA-approved IDH1 test. Olutasidenib has a boxed warning about the risk of a cytokine release–driven differentiation syndrome. The most frequent treatment-emergent adverse events are nausea, anemia, fatigue, constipation, dyspnea, pyrexia, diarrhea, and laboratory abnormalities (de Botton et al. 2023).

## Hematology

Cold agglutinin disease is a rare, chronic autoimmune disease (Roth et al. 2021). Activation of the classic complement pathway in this disease causes hemolytic crisis, acrocyanosis, thrombi, and death (Roth et al. 2021); erythrocyte transfusion is often required to reduce the symptoms of anemia in these patients. Sutimlimab-jome is a humanized monoclonal antibody targeting complement protein component 1 s subcomponent (C1s). Sutimlimab selectively prevents the activation of the classic complement pathway (Roth et al. 2021) and inhibits the hemolysis (Dhillon 2022a). Sutimlimab is an efficient, non-cytotoxic, non-immunosuppressive, and first-in-class complement inhibitor. It has been approved to decrease the requirement of erythrocyte transfusions due to hemolysis in cold agglutinin disease (Berentsen et al. 2022). Sutimlimab was granted a breakthrough therapy status following a priority review. The most frequent adverse events of sutimlimab are infection and gastrointestinal complaints (Dhillon 2022a).

Pyruvate kinase deficiency is a rare, inherited, and chronic hemolytic anemia caused by PKLR gene mutations (Al-Samkari et al. 2022). Mitapivat is a first-in-class pyruvate kinase enzyme activator and a sulfonamide derivative (Rab et al. 2021). Mitapivat restores pyruvate kinase activity and enhances hemoglobin levels via binding to pyruvate kinase tetramer allosterically (Al-Samkari et al. 2022). Mitapivat was approved for hemolytic anemia with pyruvate kinase deficiency. Mitapivat was granted a fast-track designation, priority review, and orphan drug designation. The most treatment emergent adverse events of mitapivat are sleeplessness, dizziness, and headache (Musallam et al. 2022).

Chemotherapy-induced neutropenia may occur in patients receiving myelosuppressive chemotherapy, and it is a potentially life-threating condition (Blayney and Schwartzberg 2022). Granulocyte colony stimulating factors (G-CSF)-based drugs, for example filgrastim, have been used to prevent these complications and improve prognosis (Dale et al. 2018). Eflapegrastim-xnst is a long-acting recombinant human hematopoietic growth factor (Cobb et al. 2020). Eflapegrastim contains a human G-CSF analog and a half-life-extending IgG4 Fc component linked via polyethylene glycol (Blayney and Schwartzberg 2022). Eflapegrastim stimulates signaling pathways associated with the formation, mobilization, and function of neutrophils and decreases neutropenia. Eflapegrastim has been approved for reducing the infections incidence with non-myeloid malignancies using myelosuppressive agents that have risk of febrile neutropenia. The most common adverse events associated with eflapegrastim are bone pain, arthralgia, back pain, myalgia, and headache (Schwartzberg et al. 2020).

## Neurology

The sleeping disorder insomnia is a common condition that is often accompanied by mental and physical health conditions (Sutton 2021). Daridorexant was approved for insomnia marked by disturbances of falling and/or staying asleep (Markham 2022). Daridorexant is a small molecule, orally administered, potent, selective, and competitive dual orexin type 1 and type 2 receptor antagonist (Dos Santos and da Silva 2022). Daridorexant is the third oral orexin receptor antagonist (the first is suvorexant and the second is lemborexant) approved by the FDA for the treatment of insomnia (Dos Santos and da Silva 2022). The endogenous ligands orexin A and orexin B are released in the hypothalamus and bind to orexin type 1 and type 2 receptors, which triggers wakefulness (Scammell and Winrow 2011). Antagonism of these receptors by daridorexant promotes sleep, reduces insomnia symptoms, and ameliorates sleep quality without causing addiction (Robinson et al. 2022). The most frequent adverse events of daridorexant were nasopharyngitis, headache, fatigue, dizziness, nausea, and somnolence (Markham 2022). It has been emphasized that daridorexant should not be used concurrently with other central nervous system depressants as it may increase their effects (Robinson et al. 2022).

Ganaxolone is a first-in-indication, synthetic, neuroactive steroid, and positive allosteric modulator of the γ-aminobutyric acid (GABA) A receptor (Lamb 2022a). Ganaxolone potentiates both phasic and tonic inhibition via augmenting GABAergic signaling (Vaitkevicius et al. 2022). Ganaxolone has been approved for seizures related to cyclin-dependent kinase-like 5 deficiency disorder in patients ≥ 2 years (Lamb 2022a). Ganaxolone received orphan drug designation following a priority review. Adverse reactions are sedation, somnolence, pyrexia, upper respiratory tract infections, hypersalivation, and allergy (Lamb 2022a). The risk of somnolence and sedation may increase when ganaxolone is used with central nervous system depressants (Lamb 2022a).

Sodium phenylbutyrate-taurursodiol consists of sodium phenylbutyrate A and ursodoxicoltaurine, which target neuronal degeneration pathways (Heo 2022). Sodium phenylbutyrate-taurursodiol has been approved for amyotrophic lateral sclerosis (ALS) based on a priority review and received an orphan drug designation. In the CENTAUR phase II trial (NCT03127514), it was shown to slow the ALS progression as assessed by the ALS Functional Rating Scale-Revised score (Paganoni et al. 2020). The mechanism of action is unclear, but it may involve reducing endoplasmic reticulum stress and mitochondrial dysfunction in neurons (Heo 2022). The most common adverse events are gastrointestinal complaints (Heo 2022).

Hereditary transthyretin-mediated amyloidosis (hATTR) is a progressive and rare disease with poor survival (Adams et al. 2022). Transthyretin plays a role in the transfer of vitamin A and of thyroxine into the brain and serum. Mutations of transthyretin gene cause polyneuropathy and cardiomyopathy via amyloid fibrils deposition (Ando et al. 2022). Vutrisiran is a chemically modified, transthyretin-directed small interfering ribonucleic acid (siRNA) therapeutic, which prevents amyloid production via decreasing serum and tissue-deposited transthyretin levels (Adams et al. 2022). Vutrisiran has been approved for polyneuropathy of hATTR. The most frequent adverse reactions of vutrisiran are pain, arthralgia, and transient injection-site reactions (Keam 2022d).

Ublituximab-xiiy is an antibody-based disease modifying therapy and monoclonal antibody against the CD20-expressing B-cells (Mukhtar et al. 2022). Ublituximab is glycoengineered to enhance the antibody-dependent cytotoxicity (Lee et al. 2021). Ublituximab has been approved for relapsing forms of multiple sclerosis. Ublituximab is the third approved anti-CD20 multiple sclerosis drug after ocrelizumab and ofatumumab (Mukhtar et al. 2022). Ublituximab leads to reduced B-cells via cell destruction and prevents immune system–mediated damage to the brain and spinal cord in MS (Margoni et al. 2022). The most common adverse reactions of ublituximab are infusion-related reactions (Fox et al. 2021).

## Metabolic, cardiovascular, and endocrine disorders

The cardiac myosin inhibitor mavacamten has been approved for the treatment of obstructive hypertrophic cardiomyopathy (HCM). A first-in-class medication, mavacamten, inhibits myosin heavy chain, thereby reducing the excessive actin-myosin cross-bridge formation (Ho et al. 2020). Mavacamten therapy has been demonstrated to be beneficial for exercise performance, left ventricular outflow tract (LVOT) obstruction New York Heart Association (NYHA) heart failure symptom classification and health status evaluated using the Kansas City Cardiomyopathy Questionnaire (KCCQ) (Olivotto et al. 2020; Xie et al. 2022). Mavacamten is used orally for the patients with symptomatic NYHA classes II–III obstructive HCM. It may reduce systolic function and worsen heart failure demonstrated as a decrease in ejection fraction (EF) as low as 35% in 10% of patients (Olivotto et al. 2020). Therefore, echocardiogram assessment is required before and during the treatment. Patients with EF lower than 55% are not recommended to initiate the therapy. On the other hand, clinical trials reported that the treatment with mavacamten is mostly well tolerated but adverse effects such as dizziness and syncope along with some serious side effects including acute stress cardiomyopathy, atrial fibrillation, ventricular tachycardia, and angina pectoris have been observed (Heitner et al. 2019; Olivotto et al. 2020). Mavamcamten is metabolized by CYP2C19 and CYP3A4; thus, co-administration with CYP2C19/CYP3A4 inhibitors is associated with high risk of worsening of cardiac function. Due to the risk of heart failure, mavacamten treatment is only available through a restricted program under a Risk Evaluation and Mitigation Strategy.

Tirzepatide has been approved to control glycemia and body weight through activating two hormone receptors (Chavda et al. 2022). Tirzepatide is a dual agonist of glucose-dependent insulinotropic polypeptide (GIP) and glucagon-like peptide-1 (GLP-1) receptor that are gut-derived incretin hormones. Nicknamed “twincretin,” tirzepatide is a first-in-class drug that is formulated as a synthetic peptide analog of the GIP hormone. It is administered subcutaneously once a week and was evaluated in clinical trials as a single or an add-on therapy compared to placebo, the GLP-1 receptor agonist semaglutide, or insulin analogs (Del Prato et al. 2021; Frías et al. 2021; Jastreboff et al. 2022). It results in dose-dependent decreases in HbA1_C_ levels accompanied by weight reduction. Additionally, tirzepatide was also demonstrated to improve lipid profile and blood pressure (Chavda et al. 2022). Its common adverse effects were gastrointestinal system–related, including nausea, vomiting, decreased appetite, and diarrhea. Tirzepatide is contraindicated in patients with thyroid carcinoma or multiple endocrine neoplasia syndrome type 2 since animal studies demonstrated an increase in thyroid C-cell tumor formation associated with tirzepatide treatment. Tirzepatide has been approved based on a priority review.

Type 1 diabetes (T1D) is an autoimmune disease in which insulin-producing pancreas beta cells are destroyed by T-cells. Teplizumab-mzwv is a humanized CD3-directed monoclonal antibody approved to delay the onset of the insulin-dependent, i.e., stage 3 T1D in high-risk individuals aged 8 years and older. Teplizumab is a first-in-indication drug for T1D prevention (Hirsch 2023). Teplizumab reduced the rate of progression to stage 3 T1D from 36 to 15% per year (Herold et al. 2019). Low levels of lymphocytes, rash, and headache are among its common adverse effects. Patients should be monitored for the symptoms of cytokine release syndrome, serious infections, lymphopenia, and hypersensitivity. Since teplizumab may interfere with immune response to vaccination, all age-appropriate vaccinations should be completed prior to therapy. Teplizumab received a breakthrough therapy designation following a priority review.

Hepatorenal syndrome (HRS) is a progressive kidney dysfunction in patients with advanced liver disease, for instance cirrhosis and ascites. Terlipressin, a prodrug that is converted to the endogenous hormone vasopressin (Alexander et al. 2021), is the first FDA-approved therapy to improve kidney function in HRS patients. Terlipressin has been demonstrated to improve kidney function evaluated by two consecutive days of low serum creatinine levels (Wong et al. 2021). Abdominal pain, nausea, diarrhea, and dyspnea are the common side effects of terlipressin therapy. The risk of serious or fatal respiratory failure is increased in the patients; therefore, oxygen levels should be monitored during the medication and patients with low levels of oxygen should avoid the therapy. Terlipressin may also cause fetal harm and thus must be avoided during pregnancy. It received priority review and fast track status, along with the orphan drug designation.

## Dermatology

A novel onabotulinum toxin A–based drug that inhibits acetylcholine release at the neuromuscular junction was approved in 2022: DaxibotulinumtoxinA-lanm temporarily relaxes facial muscles that cause glabellar lines and thus reduces their appearance. Compared to conventional neuromodulators, daxibotulinumtoxin A mediates a prolonged duration of glabellar line reduction response which lasts for 6 to 9 months (Bertucci et al. 2020; Carruthers et al. 2020). It is administered as an intramuscular injection into five specified sites. Daxibotulinumtoxin A is well tolerated and may cause headache, eyelid ptosis, and facial paresis as adverse reactions (Bertucci et al. 2020). The medication has also a warning for the distant spread of the toxin, which may cause swallowing and breathing difficulties.

Atopic dermatitis is an inflammatory disease that involves various immune pathways including JAK signaling, which regulates pro-inflammatory cellular response (Deeks and Duggan 2021). Abrocitinib is a JAK1 inhibitor approved to treat moderate-to-severe atopic dermatitis in adults and in children aged 12 years and older. Abrocitinib is administered orally once daily and was shown to reduce itch and atopic dermatitis better than dupilumab (Bieber et al. 2021; Reich et al. 2022). Nausea, headache, acne, and herpes simplex are among its adverse reactions. Abrocitinib is associated with increased risk of serious infections due to immune system suppression. Changes in blood levels of platelets, lymphocytes, and lipids may also be seen with abrocitinib therapy; thereby, monitoring of these parameters is recommended. Besides, live vaccines should be avoided prior to, during, or immediately after the therapy.

In 2022, three novel drugs have been approved to treat different forms of psoriasis. Tapinarof is indicated for the treatment of plaque psoriasis. It is an agonist of aryl hydrocarbon receptor, which is a ligand-dependent transcription factor playing a role in the immune system–mediated responses on the skin (Keam 2022c). A first-in-class medication, tapinarof, is administered topically once daily to reduce the severity of plaque psoriasis (Lebwohl et al. 2021). It has a favorable safety profile with common adverse reactions such as folliculitis, nasopharyngitis, contact dermatitis, headache, and pruritus (Lebwohl et al. 2021; Nogueira et al. 2022). Spesolimab-sbzo is an anti-interleukin-36 receptor monoclonal antibody that is indicated to treat generalized pustular psoriasis flares. It inhibits the inflammatory signaling of interleukin-36 that is overexpressed in pustular psoriasis (Blair 2022). It was reported to mediate higher lesion clearance than placebo and to be associated with infections and drug reactions with eosinophilia and systemic symptoms (Bachelez et al. 2021). Its most common side effects are asthenia, fatigue, nausea, headache, and pruritus (Blair 2022). Deucravacitinib has been approved for the treatment of moderate-to-severe plaque psoriasis. It is a selective tyrosine kinase 2 inhibitor that is a member of the JAK family, which is involved in immune responses and suggested as a therapeutic target for psoriasis. In contrast to other tyrosine kinase inhibitors, deucravacitinib acts through allosteric inhibition resulting in a highly selective inhibition of receptor-mediated activation of the kinase (Hoy 2022a). Deucravacitinib therapy was demonstrated to be superior to placebo and to apremilast when administered orally once daily (Armstrong et al. 2023; Strober et al. 2023). It also exerts an improved safety profile and less adverse reactions including nasopharyngitis, upper respiratory infections, sinusitis, bronchitis, rash, headache, diarrhea, herpes simplex, and acne. It may also increase the levels of blood creatine phosphokinase. Patients with active and severe infections should avoid using deucravacitinib.

Anacaulase is a mixture of proteolytic enzymes that has been approved for the removal of eschar in hospitalized adults with deep partial of full thickness burns. It received prior approval from the European Medicines Agency in 2013. It is applied topically and rapidly absorbed. In clinical studies, anacaulase reduced the need for surgery and autografting, was well as the area of burns excised and the time from injury to complete debridement following injury (Rosenberg et al. 2013). Patients may experience systemic and locase adeverse effects, including pain, hypersensitivity reactions, pruritus and pyrexia. Caution should be exercised while treating patients with coagulation disorders because anacaulase may lead to coagulopathy. An azole metalloenzyme inhibitor, oteseconazole, targeting fungal CYP51 and thereby fungal cell integrity, has been approved to reduce the incidence of recurrent vulvovaginal candidiasis in females (Hoy 2022b). It is administered orally and was shown to be effective through being noninferior to fluconazole, standard-of-care therapy of vulvovaginal candidiasis (Brand et al. 2021; Martens et al. 2022). Due to its higher selectivity towards CYP51, oteseconazole has a lower number of drug interactions based on inhibition of human CYP enzymes than ketoconazole and fluconazole. The most frequent adverse reactions associated with the therapy were headache and nausea (Martens et al. 2022). Oteseconazole is contraindicated in pregnant or lactating women and females of reproductive age.

## Other

A triple therapy consisting of vonoprazan, amoxicillin, and clarithromycin has been approved for the eradication of *Helicobacter pylori* infection. Vonoprazan is a H^+^, K^+^-ATPase inhibitor, which competitively blocks the potassium binding site. Its claimed advantages compared to other proton pump inhibitors include no requirement of acid activation, stability in an acidic environment, and independence from CYP2C19 genetic polymorphisms (Miftahussurur et al. 2020). Amoxicillin and clarithromycin have been used in other *Helicobacter pylori* eradication therapies and in numerous further indications. Safety and efficacy of this novel triple treatment were evaluated in various clinical trials and found to be superior to proton pump inhibitor–based triple therapy (Chey et al. 2022). Its frequent adverse reactions are diarrhea, stomach pain, headache, and vulvovaginal infection.

Olipudase alfa is a recombinant human acid sphingomyelinase approved in adult and pediatric patients for acid sphingomyelinase deficiency (ASMD), also called Niemann-Pick disease. It is the first approved therapy to improve symptoms non-related to the nervous system. A mutation in SMPD1 gene leads to acid sphingomyelinase deficiency and sphingomyelin accumulation in organs including lungs, liver, spleen, kidney (Keam 2022b). Olipudase hydrolyzes accumulated sphingomyelin. Olipudase was shown to be well tolerated and to significantly improve disease pathology evaluated by parameters such as liver and lung functions, and by lipid profiles. Its frequent adverse reactions were headache, cough, diarrhea, hypotension, and ocular hyperemia, and pyrexia, cough, diarrhea, rhinitis, abdominal pain, vomiting, headache, urticaria, rash, arthralgia, and pruritus in children. The levels of transaminases should be monitored prior to olipudase therapy or prior to dose escalation. Besides, it is contraindicated during pregnancy due to the risk of fetal malformations. Olipudase received fast track, breakthrough therapy, priority review, and orphan drug designations.

One of the two novel ophthalmic agents approved in 2022, faricimab-svoa, is indicated for the treatment of neovascular age–related macular degeneration and diabetic macular edema. It is a bispecific antibody that inhibits both vascular endothelial growth factor (VEGF) and angiopoietin-2 (Ang-2). VEGF-A inhibition suppresses endothelial cell proliferation, neovascularization, and vascular permeability, while Ang-2 inhibition contributes to vascular stability and desensitization of vessels to VEGF (Shirley 2022). Faricimab is administered through intravitreal injection, and it is non-inferior to aflibercept used as a comparator control (Khanani et al. 2021; Eter et al. 2022). Conjunctival hemorrhage is its most common side effect. The second ophthalmic drug approved is omidenepag. It is a relatively selective agonist of the prostaglandin E_2_ (EP2) receptor approved to reduce intraocular pressure in patients with open-angle glaucoma or ocular hypertension. Clinical trials have demonstrated that omidenepag reduces intraocular pressure by enhancing outflow without complications (Aihara et al. 2021; Matsuo et al. 2022). The most frequent adverse reactions are conjunctival hyperemia, photophobia, vision blurred, dry eye, instillation site pain, and visual impairment.

Gadopiclenol is a contrast agent that is a paramagnetic macrocyclic non-ionic complex containing gadolinium. Gadopiclenol is indicated to detect and visualize abnormal lesions in the central nervous system. It has been approved with a black box warning regarding the increases risk for nephrogenic systemic fibrosis in patients with impaired elimination, such as encountered in severe kidney diseases. Clinical trials demonstrated that gadopiclenol has a good safety profile (Hao et al. 2019; Jurkiewicz et al. 2022).

Lenacapavir is a first-in-class capsid inhibitor approved for treating multi-drug-resistant HIV-1. It is available in various application forms and results in undetectable viral loads when given in optimized combinations with other antiretroviral drugs (Gupta et al. 2023). Site reactions and nausea were the most common adverse reactions along with the site reactions such as swelling, pain, and redness (Paik 2022a). Lenacapavir received priority review, fast track, and breakthrough therapy designations.

## General trends and conclusions

The number of novel drugs approved by the FDA in 2022 (37, Table 2) was markedly lower than the average of the preceding 3 years. It is tempting to speculate that this reflects the effect of the COVID-19 pandemic, especially on the recruitment into clinical trials and on the inspections of drug manufacturing sites. However, similar fluctuations were observed in the past, including halving of drug approvals in 2016 as compared to 2015 and 2017.

**Table 2:** 2022 FDA drug approvals grouped by novelty as defined in the “Methods” section. The percentages are those of first- and next-in-class drugs with all drugs approved in 2022 taken as 100%. Where available, the International Nonproprietary Name stems in drug names have been highlighted in underlined based on information of the Stem Book (https://cdn.who.int/media/docs/default-source/international-nonproprietary-names-(inn)/inn-bio-review-2022.pdf?sfvrsn=f8db166f_3&download=true)

First-in class (54%)	Approved for	Next-in-class (46%)	Approved for
Deucravacitinib	Plaque psoriasis	Abrocitinib	Atopic dermatitis
Ganaxolone	Seizure disorder	Adagrasib	Non-small cell lung cancer
Hyperpolarized ^129^Xe	Evaluating pulmonary function and imaging	Anacaulase-bcdb	Eschar removal
Lenacapavir	*Human immunodeficiency virus* infection	Daridorexant	Insomnia
Lutetium (^177^Lu) vipivotide tetraxetan	Prostate cancer	DaxibotulinumtoxinA-lanm	Glabellar lines
Mavacamten	Obstructive hypertrophic cardiomyopathy	Eflapegrastim	Febrile neutropenia
Mirvetuximab soravtansine-gynx	Ovarian cancer	Faricimab-svoa	Neovascular aged-related macular degeneration and diabetic macular edema
Mitapivat	Hemolytic anemia	Futibatinib	Intrahepatic cholangiocarcinoma
Mosunetuzumab-axgb	Follicular lymphoma	Gadopiclenol	Detecting and visualizing lesions
Nivolumab and relatlimab-rmbw	Melanoma	Omidenepag isopropyl	Open-angle glaucoma or ocular hypertension
Olipudase alfa-rpcp	Acid sphingomyelinase deficiency	Olutasidenib	Acute myeloid leukemia
Spesolimab-sbzo	Generalized pustular psoriasis flares	Oteseconazole	Recurrent vulvovaginal candidiasis
Sutimlimab	Cold agglutinin disease	Pacritinib	Myelofibrosis
Tapinarof	Plaque psoriasis	Sodium phenylbutyrate-taurursodiol	Amyotrophic lateral sclerosis
Tebentafusp-tebn	Uveal melanoma	Tremelimumab-actl	Hepatocellular carcinoma
Teclistamab-cqyv	Multiple myeloma	Ublituximab-xiiy	Multiple sclerosis
Teplizumab-mzwv	Delay the onset of stage 3 type 1 diabetes	Vutrisiran	Hereditary transthyretin-mediated amyloidosis
Terlipressin	Hepatorenal syndrome		
Tirzepatide	Glycemic control		
Vonoprazan, amoxicillin, and clarithromycin	*Helicobacter pylori* infection		

Lower overall drug approval numbers in 2022 may have been partly compensated by a further increase in innovation. Thus, first-in-class approvals accounted for 54% of all approvals Table 2), compared to 42% in 2021 (Kayki-Mutlu et al. 2022). These drugs have mechanisms of action different from those of existing therapies.

Several other trends appear to continue in 2022 despite the lower number of newly approved drugs. One is the frequent use of accelerated approval procedures: In 2022, 32% of approved drugs were granted fast track status, 35% breakthrough therapy designation, 57% priority review, and 16% accelerated approval. The availability of these accelerated regulatory pathways may also have provided incentives to focus clinical development projects on conditions where such designations are likely. Concurrently, 54% of newly approved drugs represent first-in-class treatments, even if that does not always provide relevant clinical benefits over existing treatments. Furthermore, oncology and rare diseases remain the strongest therapeutic areas for newly approved drugs with each contributing 11 and 20 of 37 (29% and 54%) of new approvals, respectively (Table 1). This development focus of the pharmaceutical industry partly reflects medical needs and partly the incentives resulting from the U.S. 1983 Orphan Drug Act and the 2002 Rare Diseases Act. Moreover, it may also in part reflect that treatments for lethal diseases such as cancers and for orphan diseases provide more promising pricing options for pharmaceutical companies. Moreover, orphan diseases can be attractive commercially because they allow a rapid clinical development and early market entry that may be followed by a later development of the same drugs for more prevalent diseases.

Another continuing trend is the increasing share of biopharmaceuticals (biologics, mostly therapeutic antibodies), which accounted for almost every-second newly approved drug in 2022. This is the highest percentage of biologics among yearly FDA approvals to date. Among antibody-based therapeutics (Mullard 2021) (Table 3), classical monoclonal antibodies are increasingly supplemented by bispecific antibodies such as faricimab and by antibody–drug conjugates such as mirvetuximab. On the other hand, G protein–coupled receptors have been one of the most popular drug targets in the past (Sriram and Insel 2018) but apparently are becoming targeted less frequently by newly approved drugs, terlipressin being one of the few exceptions.

**Table 3:** 2022 FDA drug approvals grouped by drug type. The percentages are those of small molecule, antibody, and peptide drugs, with all drugs approved by the FDA in 2022 taken as 100%. The siRNA drug vutrisiran was not included in the table for the ease of reading

Small molecule (51%)	Antibody (27%)	Peptide (19%)
Abrocitinib	Aricimab-svoa	Anacaulase-bcdb
Adagrasib	Mirvetuximab soravtansine-gynx	DaxibotulinumtoxinA-lanm
Daridorexant	Mosunetuzumab-axgb	Eflapegrastim-xnst
Deucravacitinib	Nivolumab and relatlimab-rmbw	Olipudase alfa-rpcp
Futibatinib	Spesolimab-sbzo	Tebentafusp
Gadopiclenol	Sutimlimab-jome	Terlipressin
Ganaxolone	Teclistamab-cqyv	Tirzepatide
Hyperpolarized Xe-129	Teplizumab-mzwv	
Lenacapavir	Tremelimumab-actl	
Lutetium (177Lu) vipivotide tetraxetan	Ublituximab-xiiy	
Mavacamten		
Mitapivat		
Olutasidenib		
Oomidenepag isopropyl		
Oteseconazole		
Pacritinib		
Sodium phenylbutyrate-taurursodiol		
Tapinarof		
Vonoprazan, amoxicillin, and clarithromycin		

## Data Availability

Not applicable.
